# Chemoprevention of pancreatic cancer using solid-lipid nanoparticulate delivery of a novel aspirin, curcumin and sulforaphane drug combination regimen

**DOI:** 10.3892/ijo.2012.1636

**Published:** 2012-09-21

**Authors:** DHRUVITKUMAR SUTARIA, BALAGANGADHAR KARTHIK GRANDHI, ARVIND THAKKAR, JEFFREY WANG, SUNIL PRABHU

**Affiliations:** 1 Department of Pharmaceutical Sciences, College of Pharmacy, Western University of Health Sciences, Pomona, CA 91766;; 2 College of Pharmacy, The Ohio State University, Columbus, OH 43210, USA

**Keywords:** chemoprevention, pancreatic cancer, combination therapy, nanotechnology, solid lipid nanoparticles

## Abstract

Pancreatic cancer is the fourth largest cause of cancer deaths in the Unites States and the prognosis is grim with <5% survival chances upon diagnosis. The objective of this study was to assess the combined chemopreventive effect of solid lipid nanoparticle (SLN) encapsulated drugs aspirin (ASP), curcumin (CUR) and free sulforaphane (SFN) for the chemoprevention of pancreatic cancer. Experiments were carried out (1) to evaluate the feasibility of encapsulation of these chemopreventive agents within solid lipid systems and (2) to measure the synergistic effects of a combination of ASP with CUR in SLNs mixed with free SFN against cell proliferation and apoptosis in pancreatic cancer cells, MIA PaCa-2 and Panc-1. The SLNs were prepared using a modified solvent evaporation technique and were characterized for particle sizing, encapsulation efficiency and drug release. ASP and CUR SLNs were formulated within the particle size range of 150–250 nm and were found to have an encapsulation efficiency of 85 and 69%, respectively. Sustained release of drugs over a 96 h period from SLNs was observed. The SLNs were stable over a 3-month storage period at room temperature. Cell viability studies demonstrated that combinations of low doses of ASP SLN (25 *μ*M), CUR SLN (2.5 *μ*M) and free SFN (5 *μ*M) significantly reduced cell viability by 43.6 and 48.49% in MIAPaca-2 and Panc-1 cell lines, respectively. Furthermore, increased apoptosis of 61.3 and 60.37% was found in MIA Paca-2 and Panc-1 cell lines, respectively, in comparison to the individual doses administered. Synergistic effects were demonstrated using MTS and apoptosis assays. Thus, this study successfully demonstrated the feasibility of using a solid lipid nanoparticulate system for the first time to deliver this novel combination chemoprevention regimen, providing valuable evidence for the usability of nanotechnology-based drug regimens towards pancreatic cancer chemoprevention.

## Introduction

Pancreatic cancer is the fourth most common cause of cancer deaths in United States with a five-year survival rate of less than 5%. According to American Cancer Society, in the US alone it is estimated that 43,920 individuals will be diagnosed with and 37,390 of them will die of pancreatic cancer in 2012 (1). Pancreatic cancer arises from the morphologically and genetically clearly defined precursor lesions through a step-wise accumulation of genetic alterations. In majority of the patients diagnosed with pancreatic cancer, symptoms do not develop until it is either unresectable or metastatic, rendering it difficult to cure (2). The low survival rate of patients points towards an increased need for novel strategies to combat pancreatic cancer. The concept of chemoprevention has recently received significant attention as a novel strategy towards preventing pancreatic cancer before it occurs (3). Some chemopreventive agents such as COX-2 inhibitors, green and black tea derivatives, β-carotene, vitamins, isothiocyanates, and farnesyl transferase inhibitors have been reported as potential chemopreventive agents (4–7).

Aspirin (ASP), a well-known non-steroidal anti-inflammatory drug (NSAID), has emerged as a promising chemopreventive agent against various types of cancer. ASP is capable of suppressing pancreatic cancer growth both *in vitro* and *in vivo*(8). Cyclooxygenase-2 (Cox-2) enzyme which plays a key role in prostaglandin E2 (PGE2) synthesis is overexpressed in several cancers including pancreatic cancer. ASP has been shown to inhibit pancreatic carcinogenesis by the inhibition of Cox-2 enzymes (6). Studies associated with the use of ASP for pancreatic cancer chemoprevention have met with mixed results thus far (5,7). Given these conflicting reports on the use of ASP in pancreatic cancer, it reaffirms the need for further study of this drug in pancreatic cancer chemoprevention.

Curcumin (CUR), a diferuloylmethane is a derivative of spice turmeric (*Curcuma longa*). CUR has shown to have pronounced chemopreventive, anti-inflammatory, anti-oxidative and anti-carcinogenic activities in different cancer cell lines and murine cancer models (9–11). CUR has been shown to suppress NF-κB activation and NF-κB gene products (12) and can induce p53-dependent apoptosis by induction of p53 in certain cancer cell lines (13). In pancreatic cancer cell lines, CUR acts by suppressing the activation of NF-κB through the inhibition of IκBα protein (14).

Sulforaphane (SFN) is a naturally-occurring sulfur-containing isothiocyanate found in cruciferous vegetables such as broccoli, brussel sprouts, cauliflower and cabbage (15). SFN has been shown to reduce NF-κB activity and affect expression of NF-κB mediated genes encoding adhesion molecules, inflammatory cytokines, growth factors and anti-apoptotic factors (16). SFN also modulates multiple targets, which regulate many cellular activities including oxidative stress, apoptosis induction, cell cycle arrest, angiogenesis and metastasis suppression (17).

There is an increasing interest to use a combination of chemopreventive agents that differ in their mode of action and target multiple pathways. This approach provides a means of obtaining low-dose therapy and increased efficacy with less toxicity (18,19). It is noteworthy that the phase III clinical trial of difluoromethylornithine combination with sulindac has shown greater chemopreventive efficacy (19), which pave the way for the use of combinatorial regimens to achieve a synergistic effect. To date, no group has investigated the combined therapeutic effects of low dose mixtures of ASP, CUR and SFN on prevention of pancreatic cancer. Since all of these agents have different mechanisms of action, an anticipated synergistic effect in pancreatic cancer would provide valuable information in assessing combination chemopreventive regimens for eventual clinical use.

The method of delivery plays an important role in improving the overall drug bioavailability. Novel modes of delivery methods using microspheres and nanosphere technology are receiving wide attention as these have shown superior delivery compared to conventional dosage forms (20,21). The strength of the drug delivery system is their ability to alter the pharmacokinetics and biodistribution of the drug. Nanoparticles have unusual properties that can be used to improve drug delivery. Provided they are within the nanometer size range, there is an increased uptake by the cells through enhanced permeation and retention (EPR) effect thereby making it a potential tool to diagnose and treat cancer. Nanosized drug delivery systems offer several advantages over the conventional delivery system such as controlled and sustained release of drugs, ability of the drug to cross the mucosal barriers, decreased renal and hepatic clearance, decreased immune recognition, increased half-lives of drugs due to slow and controlled release from the nanoparticulate systems, increased stability and solubility (22). All these advantages suggest the emerging role of nanoparticles in cancer therapy and chemoprevention demonstrating a need for further research in this area.

A nanotechnology-based drug delivery system, solid-lipid nanoparticles (SLN) has received considerable attention in the last few years as a convenient method of delivering drugs into the body in a controlled release manner (23). SLNs are commonly defined as solid nanoscaled lipid matrices in size range of 50–1,000 nm typically consisting of a solid lipid compound, surfactant and incorporated active ingredients. Additionally, SLNs are biocompatible and act by protecting incorporated compounds against chemical degradation. However, the most important advantage of SLNs is that they increase the bioavailability of lipophilic drugs administered by the oral route (24). There is considerable evidence that SLNs act by carrying most of the drugs through the lymphatic system, and in part through the general blood circulation thus avoiding first pass metabolism (25). This allows for administration of lower doses with less chances of toxic effects, while maintaining efficacy. It has been observed that the formulations exhibited superior and better cytotoxicity profile compared to their corresponding free drug (26). Thus, the SLN delivery system due to the favorable physicochemical characteristics, controlled release kinetics would be ideal for delivery of lipophilic drugs like ASP, CUR and SFN.

Although many studies have been conducted to test the chemopreventive efficacy of ASP, CUR and SFN, no studies have been reported on the combined chemopreventive efficacy of these SLN encapsulated drugs. We recently demonstrated significant chemoprevention of colon cancer in rats using an orally administered drug loaded polylactide-co-glycolide (PLGA) polymer in a nanotechnology-based targeted delivery system (26). Here, we are proposing the use of a novel solid-lipid nanoparticle (SLN) technology for the oral delivery of combinations of chemopreventive agents for pancreatic cancer. Thus, in the present study we formulate the above mentioned chemopreventive agents into SLNs and further evaluate their combined chemopreventive efficacy in two different human pancreatic cancer cells, MIA PaCa-2 and Panc-1.

## Materials and methods

### Materials

For the cell culture assays and solid lipid nanoparticle (SLN) formulations, the drugs ASP, CUR and SFN were obtained from LKT Laboratories (St. Paul, MN). Dimethyl sulfoxide (DMSO) was obtained from Sigma Chemicals (St. Louis, MO). Stearic acid (lipid) and Poloxamer 188 (emulsion stabilizer) was obtained from Spectrum Chemicals (Garden, CA). Dichloromethane (DCM) was obtained from Fisher Scientific (Houston, TX).

### Human cell lines

Human pancreatic cancer cell lines MIA PaCa-2 and Panc-1 were obtained from ATCC (Rockville, MD). Cells were maintained in Dulbecco’s modified Eagle’s medium (DMEM) containing 10% fetal bovine serum (FBS) obtained from ATCC. Cells were cultured at 37°C in a humidified atmosphere of 5% CO_2_ and 95% air.

### Preparation of solid lipid nanoparticles (SLNs)

ASP and CUR SLNs were prepared using a hot melt oil-in-water (o/w) emulsion technique. Stearic acid was used as the solid lipid to make the nanoparticle formulations. Briefly, 1 mg of stearic acid was melted by heating in a water bath at 70–80°C. The drug (100 mg) was suspended in 3 ml of DCM. The suspended drug solution was then added to the melted stearic acid and heated until all DCM was evaporated. The water phase consisted of 1% poloxamer solution which was heated to the same temperature as that of the oil phase. The oil phase was then added to the poloxamer solution and the mixture was further sonicated for 5 min using an ultra-sonicator (Branson, Los Angeles, CA) to create an o/w emulsion. The emulsion so formed was then cooled and washed with water to remove excess free drug from the particle surface. SLNs were freeze-dried in a freeze dryer (Labconco, Kansas City, MO) and subjected to particle sizing and encapsulation efficiency determination.

### Particle size measurement

The particle sizes of the formulated SLNs were measured using Nicomp submicron particle size analyzer Model 370 (New York, USA). Briefly, 5 mg of the SLN formulation was suspended in 10 ml of phosphate saline buffer (PBS, pH 7.4) and was sonicated for 2 min. Particle size was measured using 1 ml of the suspension.

### Encapsulation efficiency (%) determination

Encapsulation efficiency (E.E) was determined by dissolving 10 mg of the SLN formulation in DCM which dissolves the stearic acid and releases the drug entrapped within the lipid. DCM was evaporated under a current of inert air for 1 h. Evaporation of DCM left a residue of the drug and lipid sticking to the bottom of the test tube. Drug was separated from the lipid by dissolving it in 5 ml of acetonitrile. The drug was allowed to dissolve freely for about 30 min in a bath sonicator after which it was filtered through a 0.45 *μ*m filter. The resulting solution was further diluted to 20 ml by adding acetonitrile. A total of 1 ml of the resulting mixture was analyzed using Shimadzu LC-20 binary HPLC system (Columbia, MD). A total of 20 *μ*l of naproxen was used as an internal standard. E.E was calculated using the following expression: E.E (%) = amount (mg) of drug per HPLC method/theoretical yield (mg) ×100.

### Determination of drug release from SLNs

The *in vitro* release profile of ASP and CUR from SLNs was determined using a dialysis bag method. A hydrophilic dialysis membrane pouch (MWCO 12 kDa) served as the donor compartment. SLNs containing 100 mg of the drug were suspended in 5 ml of PBS and placed inside the membrane pouch. Subsequently, the pouch was lowered into a container with 100 ml PBS solution containing 1% sodium lauryl sulfate (SLS) serving as the receptor medium for the study. At fixed time intervals, 5 ml receptor medium was withdrawn and replaced with 5 ml fresh medium. Samples were analyzed using HPLC. All the samples were carried out in triplicates.

### Drug encapsulated SLN stability studies

Storage stability studies were conducted on ASP and CUR SLN formulations over a period of 3 months. Samples were stored at three different temperatures 4°C, 24°C and 37°C in closed glass vials. Particle size and encapsulation efficiencies were determined as indicators of storage stability of the prepared SLNs. All the studies were conducted in triplicate.

### Cell viability (MTS) assay

The cell viability assay was performed according to manufacturer’s protocol included with the Promega CellTitre 96 Aqueous MTS reagent (Madison, WI). Briefly, 2.5×10^3^ cells of MIA PaCa-2 cells and 4×10^3^ cells of Panc-1 were seeded in 96-well plates and incubated for 24 h. Test compounds ASP SLNs, CUR SLNs and free SFN alone and in combination at a concentration of 25 *μ*M, 2.5 *μ*M and 5 *μ*M respectively, were added and incubated for a period of 72 h. On the last day of the incubation period, the growth medium was removed followed by addition of 100 *μ*l mixture consisting of 20% MTS and 1% of phenazine methosulfate (PMS) to the serum-free culture medium and incubated for 2 h at 37°C. MTS is bioreduced by cells into formazan which can be measured at 490 nm. Thus, the quantity of formazan product measured by the amount of 490 nm absorbance is directly proportional to the number of living cells in culture. IC_50_ values were determined using Prism software (San Diego, CA). All the samples were performed in triplicates. Each experiment was performed at least thrice.

### Annexin V-PI apoptosis assay

The assay was performed according to manufacturer’s protocol included with the Annexin V-fluorescein isothiocyanate (FITC) Vybrant Apoptosis assay kit #3 ( Invitrogen, Green Island, NY). Briefly, 3×10^5^ MIA PaCa-2 and Panc-1 cells were seeded in 6-well plates and incubated for a period of 24 h. Test compounds ASP SLNs, CUR SLNs and free SFN alone and in combination at a concentration of 25 *μ*M, 2.5 *μ*M and 5 *μ*M, respectively, were added and incubated for a period of 48 h. After the incubation period, cells were washed twice with cold PBS (phosphate buffered saline) and then resuspended in 1X Annexin binding buffer such that the cell density was equivalent to 1×10^6^ cells/ml. A total of 100 *μ*l of this cell suspension was then subjected to 5 *μ*l of FITC Annexin V and 1 *μ*l of the 100 *μ*g/ml PI followed by incubation in the dark for 15 min. After the incubation period, 400 *μ*l of 1X annexin binding buffer was added to the cells followed by gentle vortexing. The samples were kept on ice and were analyzed using Beckman Coulter Cytomics FC500 (Brea, CA). The fluorescence emission was measured at 530 nm (e.g. FL1) and >575 nm (e.g. FL3).

### Statistical analysis

Results were expressed as mean ± SEM. A one-way ANOVA followed by Tukey’s post hoc analysis using Graph pad prism software (La Jolla, CA) was done to analyze and compare the results. A probability value of ≤0.05% was considered significant.

## Results

### Particle sizing of drug encapsulated SLNs

Nicomp volume weight was used as the standard measure for poly distribution type particle sizing. The SLNs of ASP and CUR were retained within the nanometer range (Table I). The particle size of ASP SLNs (150 nm) was lower than that of CUR (249 nm). All the SLNs showed optimal particle size with low variability.

### Encapsulation efficiency (%)

The SLNs exhibited 85 and 69% encapsulation efficiency of the ASP and CUR, respectively, within the lipid (Table I). The encapsulation efficiency was higher for ASP in comparison to CUR.

### Drug release from the SLNs

**Figure 1:**
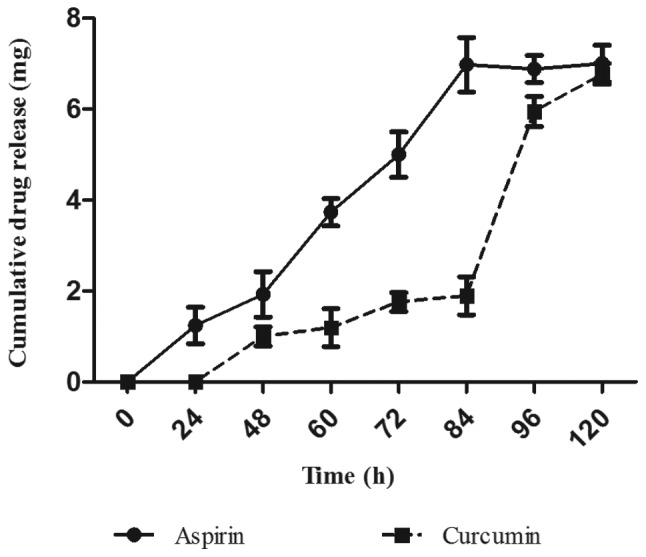
The release of aspirin and curcumin from solid lipid nanoparticles over a period of 5 days. The drug release was determined using a dialysis bag method and analysis of samples was done using an HPLC system. The data were plotted as mean ± SEM. All the samples were carried out in triplicates.

The drug release studies were performed using membrane compartmental analysis. The release of the drug from nanoparticles prepared using stearic acid as lipid was conducted over a period of 5 days. As shown in Fig. 1, the release of ASP was faster compared to CUR. Release of CUR was not observed until a period of 24 h. A cumulative drug release of approximately 7 mg of ASP was observed within 90 h of the study showing faster release pattern in comparison to the curcumin SLNs. However, in comparison to ASP SLNs, CUR SLNs showed a slower drug release time profile releasing approximately 6.5 mg of the drug entrapped. Both the formulations exhibited slow sustained release of the drug.

### Drug encapsulated SLN stability studies

**Figure 2:**
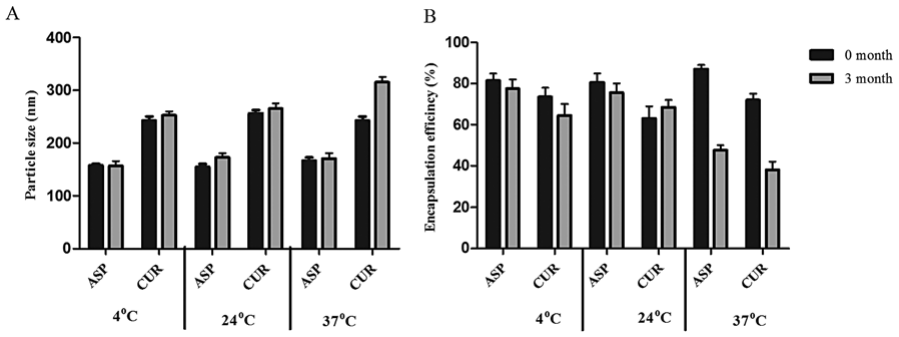
Storage stability data of aspirin (ASP) and curcumin (CUR) SLNs over a three month period at three different temperatures (4°C, 24°C and 37°C). The storage stability indicators (A) particle sizing and (B) % encapsulation efficiency were determined at start of the study and at the end of 3 month storage period. All the samples were taken in triplicates. The data were plotted as mean ± SEM.

*i) Particle size (nm).* Particle size of ASP SLNs measured at the start of the study showed a size range of 160±32 nm. After a three month duration, where samples were stored at three different temperatures (4°C, 24°C and 37°C) the particle sizes were 165, 164 and 180 nm, respectively. For CUR SLNs, the size range at the start of the storage study was 250±40 nm. At the end of 3 months study, the particle sizes were 260, 255 and 325 nm, respectively. Thus both ASP and CUR SLNs exhibited stability at lower temperatures (4°C and 24°C). However, some agglomeration was evident at 37°C which demonstrated an increase in particle size at that temperature (Fig. 2A). *ii) Encapsulation efficiency (%).* ASP SLNs showed an initial encapsulation efficiency (E.E) of 85% at the start of the storage stability test. After 3 months at different temperatures, the E.E was found to be 82% (4°C), 80% (24°C) and 45% (37°C). The encapsulation efficiency of CUR SLNs was demonstrated to be 69% but after 3 months at different temperatures showed 70% (4°C), 65% (24°C) and 34% (37°C) encapsulation (Fig. 2B). Thus, the encapsulation seemed to be affected at higher temperatures. Results of these studies demonstrated that storage at higher temperatures of 37°C could result in particle size and encapsulation changes which would have a direct adverse impact on the drug release characteristics from the SLNs used in the study.

### IC_50_ comparisons between free drugs and drug encapsulated SLNs

**Figure 3:**
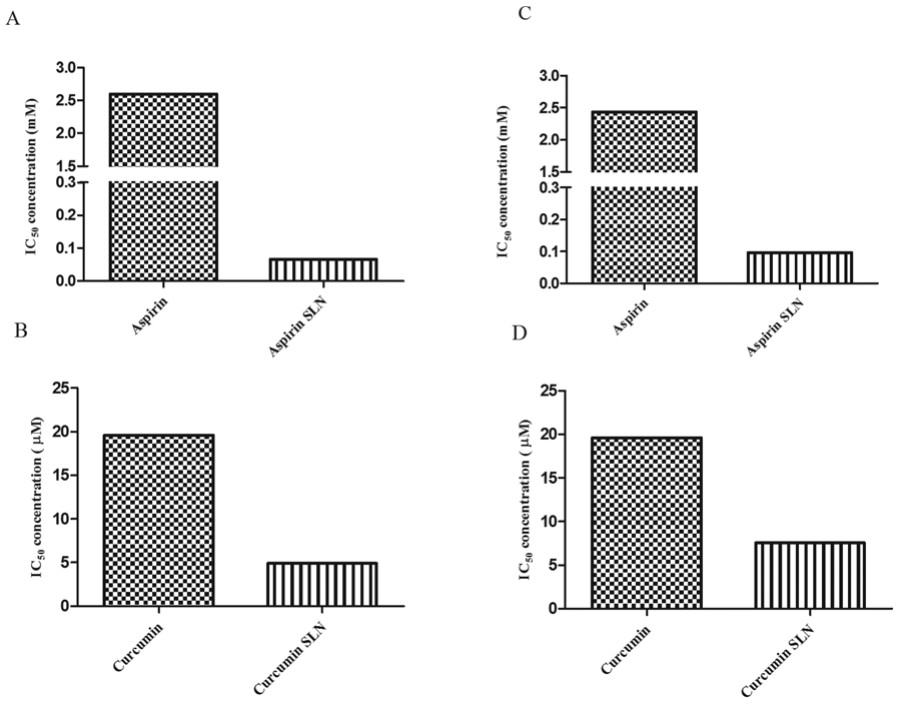
IC_50_ values comparison between free form and drug encapsulated solid lipid nanoparticles (SLNs). In MIA PaCa-2 cells, IC_50_ values were compared between free and SLN forms of (A) aspirin and (B) curcumin. In Panc-1 cells, IC_50_ comparisons were shown between free and SLN forms of (C) aspirin and (D) curcumin. MTS assay was performed to determine the cell viability of MIA PaCa-2 and Panc-1 cells after treating with a range of concentrations of free and SLN forms of aspirin and curcumin for 72 h. IC_50_ values were then determined using graphpad Prism software.

In order to study the effects of SLNs, the inhibitory concentrations (IC_50_) values of the free drug and the drug encapsulated SLNs were compared. It was observed that the SLNs exhibited lower IC_50_ values in comparison to the free drug. In case of MIA PaCa-2 cells, free ASP exhibited IC_50_ value of 2.6 mM. However, in case of drug encapsulated SLNs, the IC_50_ value was significantly reduced to 66.08 *μ*M, showing approximately 38-fold reduction compared to the free form of the drug (Fig. 3A). Whereas, free CUR exhibited the IC_50_ value of 19.6 *μ*M whereas CUR SLNs exhibited IC_50_ value of 4.93 *μ*M with ∼3-fold reductions from its free form (Fig. 3B). Similarly for Panc-1 cells, the IC_50_ values obtained for free ASP and ASP SLNs were 2.4 mM and 99.11 *μ*M, respectively (Fig. 3C). Whereas, the IC_50_ values of CUR and CUR SLNs obtained were 19.6 *μ*M and 7.569 *μ*M, respectively (Fig. 3D). Thus, our results demonstrate that the drugs when encapsulated in SLNs exhibited cytotoxicity at lower concentrations compared to free forms of the drugs.

### Novel combined chemopreventive regimen of ASP SLNs, CUR SLNs and free SFN show a synergistic effect on the reduction of cell viability

**Figure 4:**
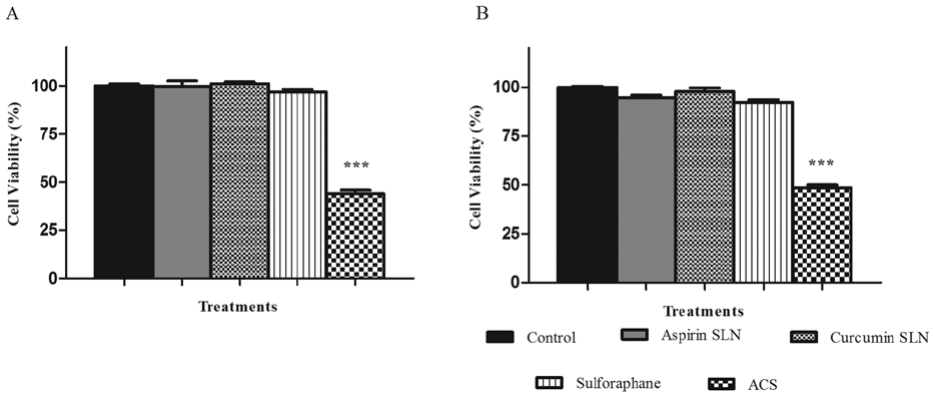
Synergistic effect of ACS combination on cell viability. MTS assay was performed to determine the cell viability of (A) MIA PaCa-2 and (B) Panc-1 cells after the treatment with aspirin SLN (ASP; 25 *μ*M), curcumin SLN (CUR; 2.5 *μ*M) and free sulforaphane (SFN; 5 *μ*M) individually and in combination of ACS (ASP+CUR+SFN) for 72 h. Each bar represents the mean percent viable cells measured in three parallel but independent experiments. Statistical significance was determined by one-way ANOVA followed by Tukey’s post hoc analysis. ^***^P<0.001 represents statistical significance of differences between control and treatment group.

MTS assay was performed in order to study the combination effects of chemopreventive drug SLNs on the cell lines MIA PaCa-2 and Panc-1. After determining the dose response curves individually and obtaining the IC_50_ value for each of them, ineffective drug concentrations were selected for ASP SLN (25 *μ*M), CUR SLN (2.5 *μ*M) showing minimal inhibitory response on the cell lines. SFN was used in its unmodified form at a concentration of 5 *μ*M. When combined together as a combination (ACS), the cell viability was reduced to 43.6% for MIA PaCa-2 cell line (Fig. 4A) and 48.49% for Panc-1 cell line (Fig. 4B), respectively. This change was significant (p<0.0001) in comparison to the reduction in cell viability of individual concentrations of ∼10%. Thus, synergistic effects were observed when combination of drugs was used. Dual combinations showed reduction in cell viability of ∼20% hence demonstrating no significant differences when compared to individual drug concentrations (data not shown).

### Combined chemopreventive regimen shows increased apoptosis in human pancreatic cancer cells

**Figure 5:**
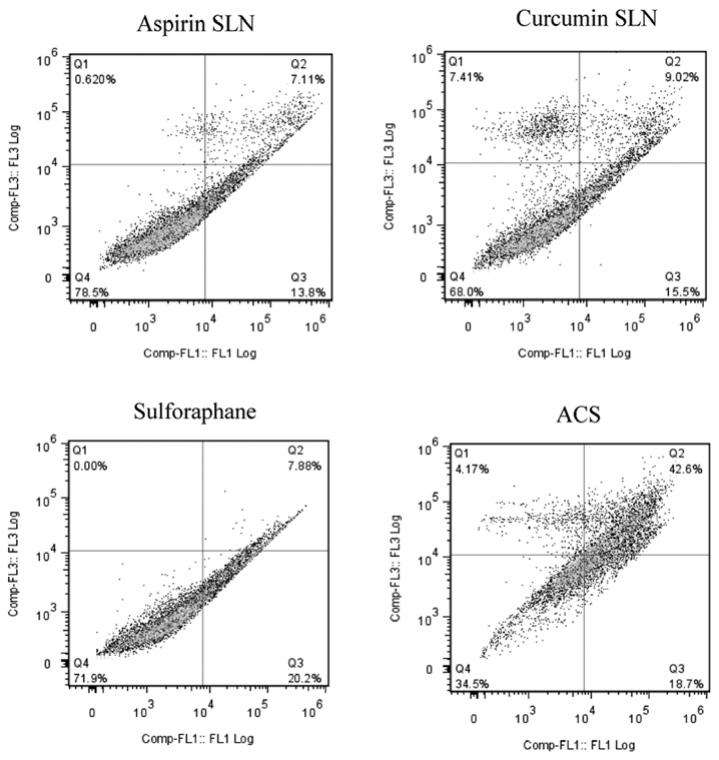
ACS combination induces apoptosis in pancreatic cancer cells. MIA PaCa-2 cells were treated with aspirin SLN (ASP; 25 *μ*M), curcumin SLN (CUR; 2.5 *μ*M) and free sulforaphane (SFN; 5 *μ*M) or in combination ACS (ASP+CUR+SFN) for 48 h. At the end of treatment, adherent and non-adherent cells were collected and stained with Annexin V-PI kit. Stained cells were then analyzed by flow cytometric analysis. The ACS chemopreventive combination demonstrated ∼61% apoptosis in MIA PaCa-2 cells.

Induction of apoptosis by ASP (25 *μ*M), CUR (2.5 *μ*M) and SFN (5 *μ*M) alone or the combination was evaluated in MIA PaCa-2 and Panc-1 cells. The cells were exposed to the drugs for 48 h, and induction of cell apoptosis was examined by Annexin-V binding and PI staining assay. In case of MIA PaCa-2 cell line as shown by the representative contour plots (Fig. 5), individual concentrations of SLN chemopreventive agents showed low evidence of apoptosis in both the cell lines; ASP SLNs exhibited 20.91%, CUR SLNs 24.52% and SFN 28.08% apoptotic cells. However, when the cells were exposed to a combination (ACS), an apoptotic effect of 61.3% was observed.

**Figure 6:**
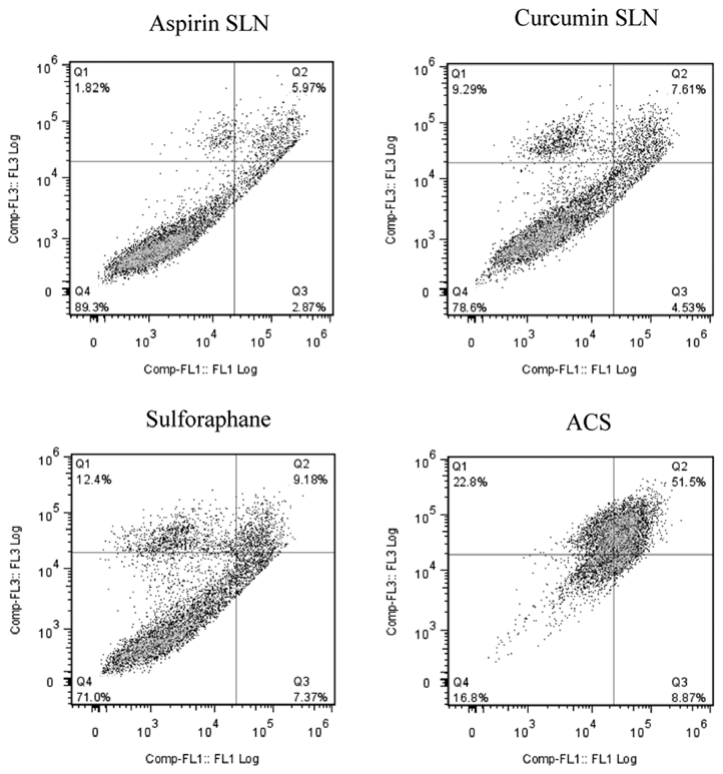
ACS combination induces apoptosis in pancreatic cancer cells. Panc-1 cells were treated with aspirin SLN (ASP; 25 *μ*M), curcumin SLN (CUR; 2.5 *μ*M) and free sulforaphane (SFN; 5 *μ*M) or in combination ACS (ASP+CUR+SFN) for 48 h. At the end of treatment, adherent and non-adherent cells were collected and stained with Annexin V-PI kit. Stained cells were then analyzed by flow cytometric analysis. The ACS chemopreventive combination demonstrated ∼60% apoptosis in Panc-1 cells.

A similar effect was observed in the case of Panc-1 cells (Fig. 6) where individual drug concentrations of ASP SLNs, CUR SLNs and SFN exhibited 8.84%, 12.14% and 16.55% of apoptotic cells, respectively. When the combination was used, increase in apoptotic effect was observed showing 60.37% apoptotic cells. Thus, the combination of SLN chemopreventive agents was synergistically effective in inducing apoptosis at low concentrations in both MIA PaCa-2 and Panc-1 cells.

## Discussion

Pancreatic cancer ranks as the fourth most deadly form of cancer in the United States with approximately 37,000 deaths each year (1). Early diagnosis of this disease is difficult because it develops without any early symptoms. Survival of patients with pancreatic cancer has been <5% over 5 years which makes this disease of great concern (2). Therapeutic outcomes with pancreatic cancer are not useful for patients especially upon a late diagnosis thus strategies to prevent this disease from occurring have become an important area of research. Recently, chemoprevention has emerged as an effective tool in the fight against various types of cancers, including colon and pancreatic cancer (19,27).

Our research has been focused on the administration of low doses of ACS combination SLNs to study its chemopreventive effects against pancreatic cancer cells MIA PaCa-2 and Panc-1. Single agent administration at low concentrations has been demonstrated to be ineffective, hence the hypothesis that two or more chemopreventive agents when delivered at low concentrations together, may exhibit an additive or synergistic effect against the cancer cells was tested. This can be attributed to the multi-factorial nature of carcinogenesis wherein cancer occurs as a result of multiple cellular changes during a prolonged time period. Of the various chemopreventive agents being studied, ASP, CUR and SFN have been proven to be effective in the chemoprevention of pancreatic cancer (2–4). Several *in vivo* and *in vitro* studies have shown that NSAIDs like aspirin and celecoxib have helped prevent the progression of pancreatic cancer. CUR and SFN, both being effective and non-toxic in nature, have been investigated for their chemopreventive actions (9,12,17). Therefore using a multi-disciplinary approach, this study investigated the synergistic effects of SLN combinations of chemopreventive agents, namely, ASP in combination with CUR and SFN.

Nevertheless, the clinical translation of these agents has been significantly hampered due to various reasons including poor oral bioavailability after administration. Engineering an SLN drug delivery system for these agents offers a means of increasing the bioavailability in the plasma and tissues in comparison to the free form of these drugs, thereby ultimately improving the therapeutic efficacy. Also, encapsulating the drug within the lipid matrix allows for administration of lower doses of drugs with less chance of toxic effects, while maintaining efficacy. Furthermore, using combination of agents which differ in their mode of action helps to obtain a desired preventive effect, while minimizing the dose concentration and its toxic effects (26). In terms of formulation development, both ASP and CUR SLNs were found to have optimal particle sizes, high encapsulation efficiency with good stability at or below room temperature. The improved stability may be explained by the use of organic solvent in the preparation process which may have improved the hygroscopic nature of the lipid. The high encapsulation can be attributed to the lipophilic nature of both ASP and CUR. Also the particle sizing was found to be in nanometer range suggesting a better chance of cellular uptake of the drugs. The formulated SLNs are non-toxic because they are made of physiological lipids such as stearic acid.

The effect of these agents was initially evaluated by calculating the IC_50_ values and then by combining the ineffective concentrations to exhibit an additive or synergistic effect against the cancer cells proving to be more efficacious at lower concentrations. A comparative study between the two forms of the drugs i.e., the free form and SLN form was carried out. It was observed that ASP and CUR SLNs IC_50_ concentrations exhibited approximately 38- and 3-fold reductions, respectively in comparison to the free form of the drug. Studies have been reported where drug loaded SLNs have exhibited better cytotoxicity profile in comparison to the free drug (28). This has been mainly attributed to the smaller particle size of the nanoparticles which increases the overall uptake of the drug. The surfactant used in the development process determines the inhibitory effect on the cells. We used Poloxamer 188 which has previously been shown to target cancerous cells, due to differences in the membrane of these cells when compared to the non-cancer cells. Poloxamer has not only been shown to inhibit multiple drug resistance (MDR) proteins and other drug efflux transporters on the surface of the cancer cells but also shown to enhance protoapoptotic signaling and decrease anti-apoptotic defense in MDR cells (29). The MTS assay on drug entrapped SLNs was carried out using ASP (25 *μ*M), CUR (2.5 *μ*M) and SFN (5 *μ*M) as individual concentrations. Individually they showed little or no decrease in the cell viability in the two cell lines, but when combined, a significant reduction by 60% was observed in MIA PaCa-2 and Panc-1 cells. In order to validate the efficacy of the combination regimen, apoptosis assay was conducted which determined the progression of a cancer cell from four different phases after the addition of the drug; living cell, early apoptotic cell, late apoptotic cell and necrotic cells. These results seem to be consistent with our findings in the MTS assay. The possible mechanisms involving the significant change for the combination could be an additive effect of the COX-2 enzyme inhibition, the regulation of the P53 suppressor pathway and by the modulation of the Nrf2 pathway; however additional studies need to be done to verify the findings.

From these results, we believe that chemoprevention is an effective way to prevent pancreatic cancer especially as the disease cannot be diagnosed at an early stage. Using a multi-disciplinary approach, this study investigated the synergistic effects of a combination of ASP and CUR SLN with free SFN. We demonstrated for the first time that this SLN combination showed a synergistic inhibition of cell viability and induce apoptosis in human pancreatic cancer cell lines. However, further *in vivo* studies have to be conducted to test the efficacy of this SLN combination. In conclusion, the results obtained from formulation studies and cell based assays clearly demonstrate the scope of developing the combination drug encapsulated SLN formulations to prevent pancreatic cancer.

## Figures and Tables

**Table I:** Particle size and encapsulation efficiency of drug-loaded solid lipid nanoparticles.

Drug	Particle size (nm)	Encapsulation efficiency (%)
Aspirin	150±63	85±5.2
Curcumin	249±65	69±3.0
